# Glucose challenge metabolomics implicates medium-chain acylcarnitines in insulin resistance

**DOI:** 10.1038/s41598-018-26701-0

**Published:** 2018-06-06

**Authors:** Christoph Nowak, Susanne Hetty, Samira Salihovic, Casimiro Castillejo-Lopez, Andrea Ganna, Naomi L. Cook, Corey D. Broeckling, Jessica E. Prenni, Xia Shen, Vilmantas Giedraitis, Johan Ärnlöv, Lars Lind, Christian Berne, Johan Sundström, Tove Fall, Erik Ingelsson

**Affiliations:** 1 Department of Medical Sciences, Molecular Epidemiology and Science for Life Laboratory, Uppsala University, Uppsala, Sweden; 2 Department of Neurobiology, Care Sciences and Society, Karolinska Institutet, Huddinge, Sweden; 3 Analytic and Translational Genetics Unit, Massachusetts General Hospital, Boston, MA United States of America; 4 Program in Medical and Population Genetics, Broad Institute of MIT and Harvard, Cambridge, MA United States of America; 5 Stanley Center for Psychiatric Research, Broad Institute of MIT and Harvard, Cambridge, MA United States of America; 6 Department of Medical Epidemiology and Biostatistics, Karolinska Institutet, Stockholm, Sweden; 7 Proteomics and Metabolomics Facility, Colorado State University, Fort Collins, CO United States of America; 8 Centre for Global Health Research, Usher Institute of Population Health Sciences and Informatics, University of Edinburgh, Edinburgh, UK; 9 Department of Public Health and Caring Sciences, Geriatrics, Uppsala University, Uppsala, Sweden; 10 School of Health and Social Studies, Dalarna University, Falun, Sweden; 11 Department of Medical Sciences, Cardiovascular Epidemiology, Uppsala University, Uppsala, Sweden; 12 Department of Medical Sciences, Clinical Diabetology and Metabolism, Uppsala University, Uppsala, Sweden; 13 Department of Medicine, Division of Cardiovascular Medicine, Stanford University School of Medicine, Stanford, CA United States of America; 14 Stanford Cardiovascular Institute, Stanford University, Stanford, CA 94305 USA

## Abstract

Insulin resistance (IR) predisposes to type 2 diabetes and cardiovascular disease but its causes are incompletely understood. Metabolic challenges like the oral glucose tolerance test (OGTT) can reveal pathogenic mechanisms. We aimed to discover associations of IR with metabolite trajectories during OGTT. In 470 non-diabetic men (age 70.6 ± 0.6 years), plasma samples obtained at 0, 30 and 120 minutes during an OGTT were analyzed by untargeted liquid chromatography-mass spectrometry metabolomics. IR was assessed with the hyperinsulinemic-euglycemic clamp method. We applied age-adjusted linear regression to identify metabolites whose concentration change was related to IR. Nine trajectories, including monounsaturated fatty acids, lysophosphatidylethanolamines and a bile acid, were significantly associated with IR, with the strongest associations observed for medium-chain acylcarnitines C10 and C12, and no associations with L-carnitine or C2-, C8-, C14- or C16-carnitine. Concentrations of C10- and C12-carnitine decreased during OGTT with a blunted decline in participants with worse insulin resistance. Associations persisted after adjustment for obesity, fasting insulin and fasting glucose. In mouse 3T3-L1 adipocytes exposed to different acylcarnitines, we observed blunted insulin-stimulated glucose uptake after treatment with C10- or C12-carnitine. In conclusion, our results identify medium-chain acylcarnitines as possible contributors to IR.

## Introduction

Impaired responsiveness to insulin, insulin resistance (IR), can lead to type 2 diabetes (T2D)^1,2^, myocardial infarction and stroke^3,4^. The pathophysiology of IR is incompletely understood and the rising global number of persons at risk of T2D and cardiovascular disease (CVD) due to IR demands new insights for prevention and treatment. Metabolic challenges, like the oral glucose tolerance test (OGTT), can reveal early pathogenic mechanisms of IR not apparent in fasting assessment^5–8^.

Untargeted plasma metabolomics quantifies a broad spectrum of small (<1,500 Da) circulating molecules and captures an integrative perspective of genomic, post-transcriptional and environmental effects^9^. Metabolomics has been used to discover fasting biomarkers for IR^10^ and T2D^11^, and has highlighted metabolic challenge profiles in small studies of healthy adults^5,6^. Using ultraperformance liquid chromatography-time-of-flight mass spectrometry (UPLC-TOF-MS) in fasting plasma from individuals from the general population, we previously found evidence for a causal effect of IR on monounsaturated fatty acid (FA) concentrations^12^, and for shared genetic origins between T2D and the metabolism of bile acids and phospholipids^13^.

Studying the challenged metabolic state is important, as insulin acts mainly in the post-prandial state and metabolic abnormalities could be masked in the fasting state. There is, however, a lack of human studies combining a metabolic challenge with repeated plasma metabolomics and gold standard assessment of IR, such as the hyperinsulinemic-euglycemic clamp (HEC) method. Ho and colleagues^7^ measured 110 metabolites in pre-/post-OGTT plasma in 377 non-diabetic persons, but used the fasting surrogate measure homeostasis model assessment IR^14^ to dichotomize the continuous trait IR. They found blunted excursions in lactate, β-hydroxybutyrate, isoleucine and pyridoxate in insulin resistant individuals, but did not study acylcarnitines or FA subtypes.

Here, we combine plasma metabolomics at three time-points during an OGTT (reflecting metabolite trajectories post glucose challenge) with HEC assessment (on a separate day, reflecting whole-body insulin sensitivity in steady state) in 470 non-diabetic 71-year-old Europeans to identify metabolite trajectories associated with IR, followed by studies in adipocytes. To our knowledge, this is the largest study combining these unique techniques to date.

## Materials and Methods

### Study approval

All participants provided written informed consent. The study was approved by the Regional Ethical Review Board of Uppsala University (251/90) and has been carried out in according with the principles of the Declaration of Helsinki as revised in 2008.

### Study population

The Uppsala Longitudinal Study of Adult Men (ULSAM) was initiated in 1970–1973 and enrolled 2,322 (81.7%) of all 2,841 men born between 1920–1924 and resident in Uppsala county, Sweden^15^. Biochemical and medical assessment at baseline and repeat assessments have been detailed online (http://www.pubcare.uu.se/ulsam/) and described previously^16^. The current study used data and biological samples obtained at the age of 71 years between August 1991 and May 1995. At this examination, the participants underwent an OGTT and HEC assessment. Due to an unfortunate freezer failure in the early 2000s, about half of the stored biological samples were lost. For the present study, we performed untargeted metabolomics in all individuals with remaining samples from the 0 min, 30 min and 120 min time points of the OGTT. Out of n = 626 individuals with OGTT metabolomics data, n = 548 individuals were included after removal of individuals with missing data for HEC and/or samples that failed metabolomics quality control. Another 78 participants were excluded because of prevalent T2D, defined as follows: self-reported diagnosis of diabetes, previous diagnosis of T2D in the Swedish Hospital Discharge Register (International Classification of Diseases [ICD] codes, 250.00 or 250.02 [ICD-9] or E11 [ICD-10]), prescription of insulin/anti-diabetic medication (Anatomical Therapeutic Chemical code, A10), fasting plasma glucose ≥7 mmol/L (126 mg/dL), or 2h-OGTT plasma glucose ≥11 mmol/L (198 mg/dL). After these exclusions, 470 individuals were eligible for the present investigation (Supplementary Fig. 1).

### OGTT and blood sampling

Overnight fasted (>8 h) individuals underwent standard 75 g oral glucose solution OGTT with venous blood sampling in EDTA tubes at baseline, 30 min, 60 min, 90 min and 120 min. A portion of each sample was spun down, stored on ice for a maximum of 4 h, and stored as plasma samples at -70 °C until analysis. For the purpose of the present study, we performed untargeted metabolomics in samples from three of the time points: 0 min, 30 min and 120 min.

### HEC

The method by DeFronzo *et al*.^17^ was modified with a slightly higher insulin infusion dose (56mU/min per body surface area, where DeFronzo *et al*. used 40 mU) to more thoroughly suppress hepatic gluconeogenesis^18^ and carried out separate from OGTT after about one week. Participants were instructed to attend after an overnight fast and all participants were assessed with the same clamp protocol at Uppsala University Hospital. No standardized meal was prepared on the day before the clamp procedure. Following placement on a warmed blanket, intravenous cannulation of the preferred forearm was performed in the antecubital fossa for infusions and the dorsum of the hand for blood sampling. After 40 min of rest, a baseline blood sample was taken and a bolus injection of semisynthetic human insulin was given over 10 min, followed by continuous infusion (56 mU × min^−1^ × m^2^ × body surface area^−1^) for 110 min. Steady-state glycemia of 5.1 mmol/L (91.8 mg/dL) was achieved by titration of a 20% glucose infusion and GlucAnalyzer readings every 5 min. Steady-state estimates were obtained as the mean value between 60–120 min and the glucose disposal index M was derived as the amount of glucose taken up during the 60 min of the stead-state condition. The insulin sensitivity index M/I (mg × kg^−1^ × kg × body weight^−1^ × min^−1^ per mU/L × 100) was calculated by dividing the glucose disposal index M by the mean insulin concentration during the corresponding period. The M/I index thus represents a measure of tissue sensitivity to insulin per unit of insulin, i.e. the amount of glucose metabolized per unit of insulin. The Pearson correlation coefficient between glucose disposal (M value) and the M/I index was 0.93 in our study sample. In secondary analyses, we replicated top associations using M value instead of M/I as outcome (Supplementary Methods). Estimating total body insulin sensitivity in the described setting assumes complete suppression of endogenous glucose production; and under euglycemic condition, about 90% of hepatic glucose production is suppressed by increasing insulin concentrations to 60 mU/L^18^.

### UPLC-TOF-MS

Venous blood samples collected in 1991–1995 were kept on ice for a maximum of 4 h, spun down and stored as EDTA plasma samples for ~20 years at -70 °C. The time delay was inevitable as the metabolomics methods used in this study only became available in the 2000s and biobank samples were used for analysis. Signal processing and statistical analyses were adjusted for storage time as explained below. Thawed EDTA plasma samples were protein-precipitated in methanol and analyzed on a Waters *Acquity UPLC* and *Xevo G2-TOF* spectrometer. Data were acquired at 5 Hz in positive electrospray ion mode at 6 V and 15–30 V with m/z range 50–1,200. Data processing by *XCMS* is detailed in the Supplementary Materials and (https://github.com/andgan/metabolomics_pipeline)^19^. A total of 10,162 features were detected, outliers removed, and features were annotated by matching retention time and m/z to in-house standards or public reference libraries according to Metabolomics Standard Initiative guidelines^19^. Signals were adjusted for factors of unwanted variability (retention time shift, analysis date, sample collection and plate effect) by ANOVA-type normalization. Metabolomics data are available in Metabolights, accession number MTBLS124 (http://www.ebi.ac.uk/metabolights/).

### Statistical analysis of human data

Log_2_-scaled metabolite signal intensities (*Met*) were analyzed by linear regression as follows: First, M/I = *Met*_*0*_ + *Met*_*30*_ + *Met*_*120*_ + age, was tested against M/I = age, by ANOVA likelihood ratio test, and metabolites that explained more variation in IR than the age-only model at *P* < 2.6 × 10^−4^ (Bonferroni-correction for 192 comparisons) were taken forward. Second, for metabolites taken forward from step one, M/I = Δ(*Met*_0_ - *Met*_30_) + Δ(*Met*_30_ - *Met*_120_) + age, was tested at *P < *1.4 × 10^−3^ (35 comparisons) for associations between change in plasma signal intensity during OGTT and IR. Supplementary Fig. 1 illustrates a summary of the methods. We ascertained normal distributions in histograms, QQ-plots and residual-against-fitted-value plots. Outliers and leverage were assessed in boxplots and plots of Cook’s distance and studentized residuals. All models additionally adjusted for sample quality as metabolomics analysis was performed after a ~20 yr delay between sampling in the 1990s and UPLC-TOF-MS analysis in the 2010s when the new technology became available. It has been shown that freezer storage time and freezing cycles can affect metabolite measurements^20^ and we accounted for these factors of unwanted variability as follows: Biobank logbooks for deep-frozen plasma covering the entire storage period were scrutinized for entries on sample quality or freezing cycles and analyses were adjusted by storage time in days, comments indicating possible previous thawing and re-freezing (dummy variable, 122 of 470 samples included in the present study; 26%) and any logbook comments querying possible hemolysis (dummy variable, 8 of 470 samples, 2.7%). The study was underpowered to assess associations in the 78 persons with prevalent T2D who were excluded.

### Murine 3T3-L1 preadipocyte culture and differentiation

The mouse preadipocyte line 3T3-L1 was obtained from ATCC and cultured according to the manufacturer’s instructions in high glucose (4.5 g/L) Dulbecco’s Modified Eagle’s Medium (DMEM) (Invitrogen; Carlsbad, CA) supplemented with 10% fetal bovine serum (FBS), 100 U/mL penicillin and 100 μg/mL streptomycin. Differentiation was initiated in 48 h post-confluent cells plated on 24- or 96-well plates for lipolysis and glucose uptake assays, respectively, with 0.5 mM 3-isobutyl-1-methylxanthine (Sigma), 0.5 μM dexamethasone (Sigma), and 1.25 μM human insulin (Sigma) in DMEM-10%-FBS. After 2 d, the differentiation medium was replaced with DMEM-10%-FBS supplemented by 1 μg/mL insulin for another 2 d. Thereafter, cells were maintained in DMEM-10%-FBS that was replaced every 2 d. Experiments were performed 10–14 d after differentiation initiation. The degree of differentiation was close to 100% for all experiments and did not differ between wells within plates. For experiments, overnight serum-starved, fully differentiated adipocytes were used.

### Tests for cell culture contamination

We excluded contamination by mycoplasma through Hoechst indirect DNA staining of the starter culture used for all experiments. Mycoplasma contamination thereafter would also have been detected, since all experimental wells were Hoechst-stained and imaged using the DAPI fluorescence microscopy channel following glucose uptake and lipolysis testing in order to count cell nuclei. Bacterial and fungal contamination was excluded visually by inspecting different levels of the supernatant in each well by light microscopy.

### Glucose uptake assay

Insulin-stimulated glucose uptake was measured as previously described^21^ using the fluorescently labeled glucose analog 2-deoxy-2-[(7-nitro-2,1,3-benzoxadizol-4-yl) amino]-D-glucose (2-NBDG) (Thermo Fisher) in fully differentiated 3T3-L1 cells plated on black 96-well plates with clear bottoms (Corning). 3T3-L1 adipocytes were serum-starved overnight in high-glucose DMEM (4.5 g/L). Cells were pre-incubated with or without different acylcarnitines (100 µM) for 2 h. After the first hour of incubation, cells were washed twice with PBS and glucose-starved for another hour in glucose-free DMEM using the same acylcarnitine treatments. This was followed by 20 min incubation with or without insulin (100 nM) at 37 °C in 100 μL DMEM (1 g/L glucose) containing 100 μg/mL 2-NBDG. Cells were then washed three times in 150 μL phosphate-buffered saline (PBS) before addition of 100 μL PBS containing Hoechst nuclear staining solution (Thermo Fisher) at a final concentration of 1.83 μL/mL. Intracellular fluorescence was quantified by computer-assisted microscopy (EVOS FL Auto Cell Imaging System, Life Technologies) using the GFP channel for 2-NBDG fluorescence intensity and the DAPI channel to image Hoechst-stained cell nuclei. Microscopy images (10 × magnification, 16 images/well) were saved and exported for analysis using in-house scripts in CellProfiler (v2.2.0)^22^ and Python. Analysis scripts are available on request. In brief, the software identifies individual cells in each image, quantifies the difference in fluorescence signal in cells compared to the background, and calculates average intracellular fluorescence signals adjusted for the number of cells per image. Experiments in n = 3 wells were performed in replicates.

### Lipolysis assay

Differentiated, overnight serum-starved 3T3-L1 adipocytes were pre-incubated in high-glucose DMEM (4.5 g/L) with 2% fatty acid-free BSA containing 100 µM of different acylcarnitines (acetyl-DL-carnitine HCL (C2), decanoyl-L-carnitine (C10), lauroyl-L-carnitine (C12) and palmitoyl-L-carnitine (C16; all obtained from Sigma), followed by addition of 100 nM isoproterenol with or without 10 nM insulin for 1 h. Free glycerol levels present in the medium were determined using a colorimetric kit (Free Glycerol reagent; Sigma-Aldrich) and normalized to cell density. In short, 20 μL medium/well was mixed with 100 μL glycerol reagent, incubated for 15 min at room temperature and absorbance measured at OD 540 nm using the Varioskan LUX multimode microplate reader (Thermo Scientific). Cells were stained with Hoechst nuclear staining solution (Thermo Fisher, final concentration 1.83 μL/mL) and cell number/well was quantified using computer-assisted microscopy (see ‘Glucose uptake’ for details) for normalization of free glycerol concentration by cell density. Experiments in n = 2 wells were performed in triplicates.

### **Statistical analysis of*in vitro*results**

*In vitro* glucose uptake and lipolysis results were assessed for normality by histogram and Shapiro-Wilk test. This implied no significant aberration from normal distribution, both for raw data and ANOVA residuals including main effects for treatment (insulin or isoproterenol and isoproterenol-insulin for glucose uptake and lipolysis, respectively), condition (acylcarnitines) and interaction effects. Two-sample t-tests (two-tailed) were used to compare the effect of added insulin between acylcarnitine conditions. Unadjusted *P*-values are reported. Analyses were performed in R v.3.2.2.

### Data availability statement

Full raw metabolomics data are available by open access in the MetaboLights archive, accession number MTBLS124 (http://www.ebi.ac.uk/metabolights/). The source code of the metabolomics bioinformatics pipeline is available by open access online (https://github.com/andgan/metabolomics_pipeline/) and explained elsewhere^18^. Individual-level phenotype data from ULSAM are not deposited in the public domain as existing ethical permits do not allow this. Full datasets are made available to researchers who meet the criteria for confidential data access as stipulated by participant informed consent and institutional review board permission at Uppsala University. Data access is granted through the Interdisciplinary Collaboration Team on Uppsala Longitudinal Studies (ICTUS; http://www.pubcare.uu.se/ulsam/; contact: vilmantas.giedraitis@pubcare.uu.se).

## Results

**Figure 1:**
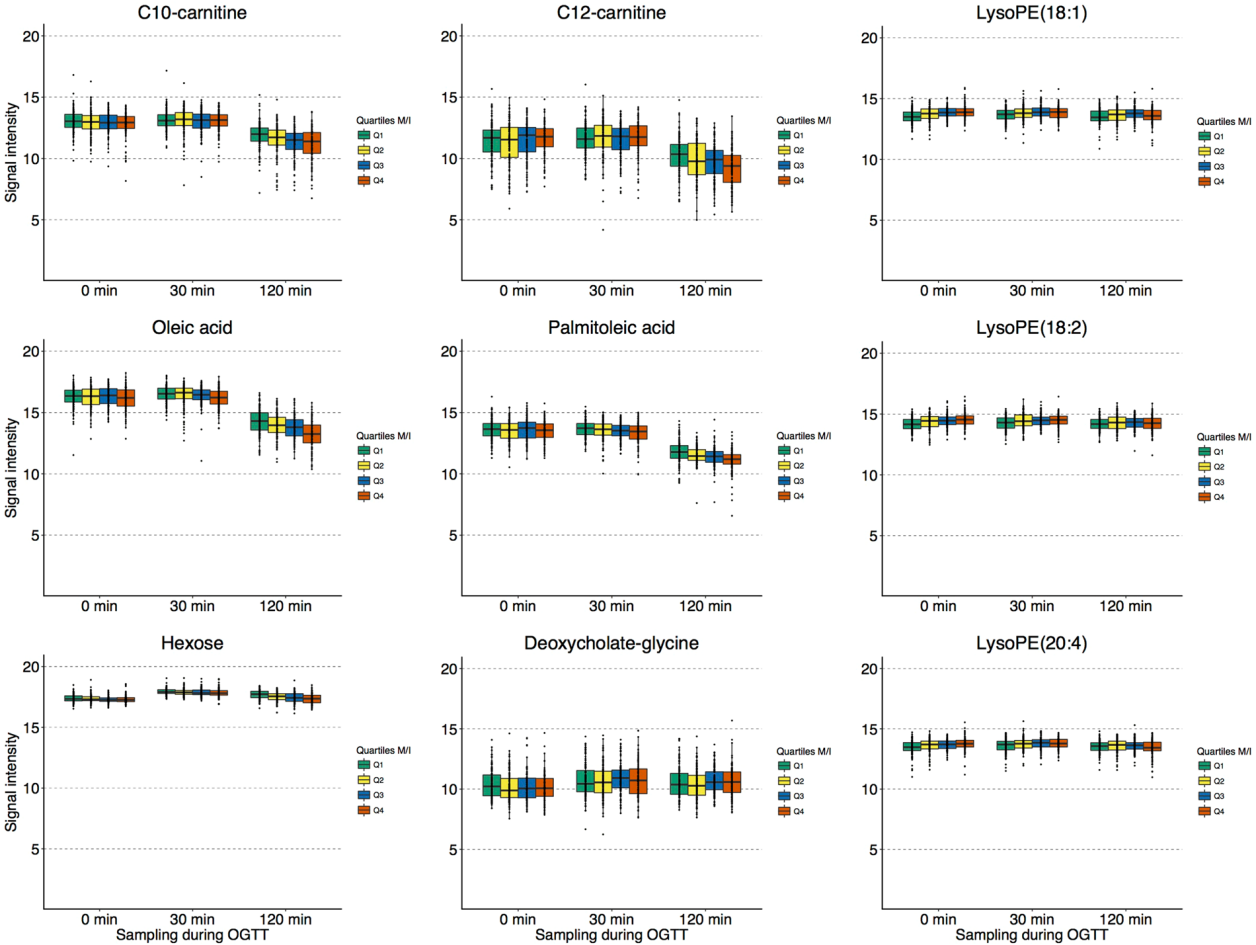
Abundance of plasma metabolites during OGTT according to insulin sensitivity. UPLC-TOF-MS signal intensity normalized for factors of unwanted variability is plotted on log_2_-scale in *n* = 470 non-diabetic men grouped according to quartile of insulin sensitivity (M/I) in boxplots. Quartile 1 (green) represents highest IR; quartile 4 (red) the least IR.

We studied 470 non-diabetic European men (age, 70.6 ± 0.6 yr, Table 1) who underwent HEC assessment and 2-h-OGTT with blood sampling at 0 min, 30 min and 120 min (Supplementary Fig. 1) on separate days (about one week apart). Untargeted UPLC-TOF-MS plasma analysis and processing through an in-house open-access bioinformatics pipeline^19^ enabled annotation of 192 metabolites included in the present study. We applied age- and sample quality-adjusted linear regression with HEC M/I ratio (glucose disposal per unit of infused insulin) as outcome. First, we discovered 35 metabolites that were associated with IR at the Bonferroni-adjusted significance level in linear models combining all three time-points during OGTT. These included seven glycerophospholipids, six glycerolipids, four glycerophosphoethanolamines, six unsaturated FAs, four acylcarnitines, two bile acids, and one each of monosaccharides, peptides, saturated FA, steroids, imidazopyrimidine and propranolol (Supplementary Fig. 1). The regression results for all 35 out of 192 tested metabolites that were significantly associated with IR at either one or several time points are listed in Supplementary Table 1. Second, we studied associations between M/I and trajectories of these 35 metabolites (Δ0–30 min and Δ30–120 min) and identified nine trajectories associated at the Bonferroni-adjusted level (listed in Supplementary Table 2). In order to illustrate the complex associations, we depict in Fig. 1 the metabolite levels during OGTT according to M/I quartile. The declines of oleate, palmitoleate, C10-carnitine and C12-carnitine levels were blunted in individuals with IR. Lysophosphatidylethanolamine (LysoPE) 18:1, LysoPE 18:2 and LysoPE 20:4 levels showed little change during OGTT in the most insulin resistant men, but decreased from higher fasting values in insulin sensitive participants. Deoxycholate-glycine levels rose during 0–30 min from a higher baseline in more insulin resistant individuals. As expected, hexose (reflecting mainly glucose) levels rose in all subjects and declined to baseline in insulin sensitive persons, with a blunted decline to above baseline in IR. Our findings replicate OGTT metabolite profiles in 16 healthy adults^23^, who also showed declines in plasma medium-chain acylcarnitines and monounsaturated FAs as well as an early-phase rise in bile acids. We now report that these trajectories are blunted in IR in a ~30-fold larger sample.

**Table 1:** Characteristics of 470 non-diabetic men included in the present study (mean ± SD or proportion).

Trait	
M/I^A^	5.3 ± 2.4
Fasting glucose (mmol/L)	5.4 ± 0.6
Fasting insulin (mU/L)	13.6 ± 6.6
OGTT glucose (AUC)	56.0 ± 30.0
OGTT insulin (AUC)	1225.9 ± 749.5
Current smoker^B^	20.1%
At least moderate physical activity	88.3%
BMI (kg/m^2^)	26.0 ± 3.3
Waist circumference (cm)	93.2 ± 9.5
Systolic BP (mmHg)	144 ± 19
Diastolic BP (mmHg)	82 ± 10
Antihypertensive medication	30.2%
Serum triglycerides (mmol/L)	1.4 ± 0.7
Total serum cholesterol (mmol/L)	5.9 ± 1.0
HDL-cholesterol (mmol/L)	1.3 ± 0.4
Lipid medication^B^	10.2%
C-reactive protein (mg/L)	3.3 ± 4.7

^A^HEC M/I in mg × kg^−1^ × kg BW^−1^ × min^−1^ per mU/L × 100.

^B^Missing responses (3.3% for smoking, 1.7% for lipid medication) counted as “no”.

**Figure 2:**
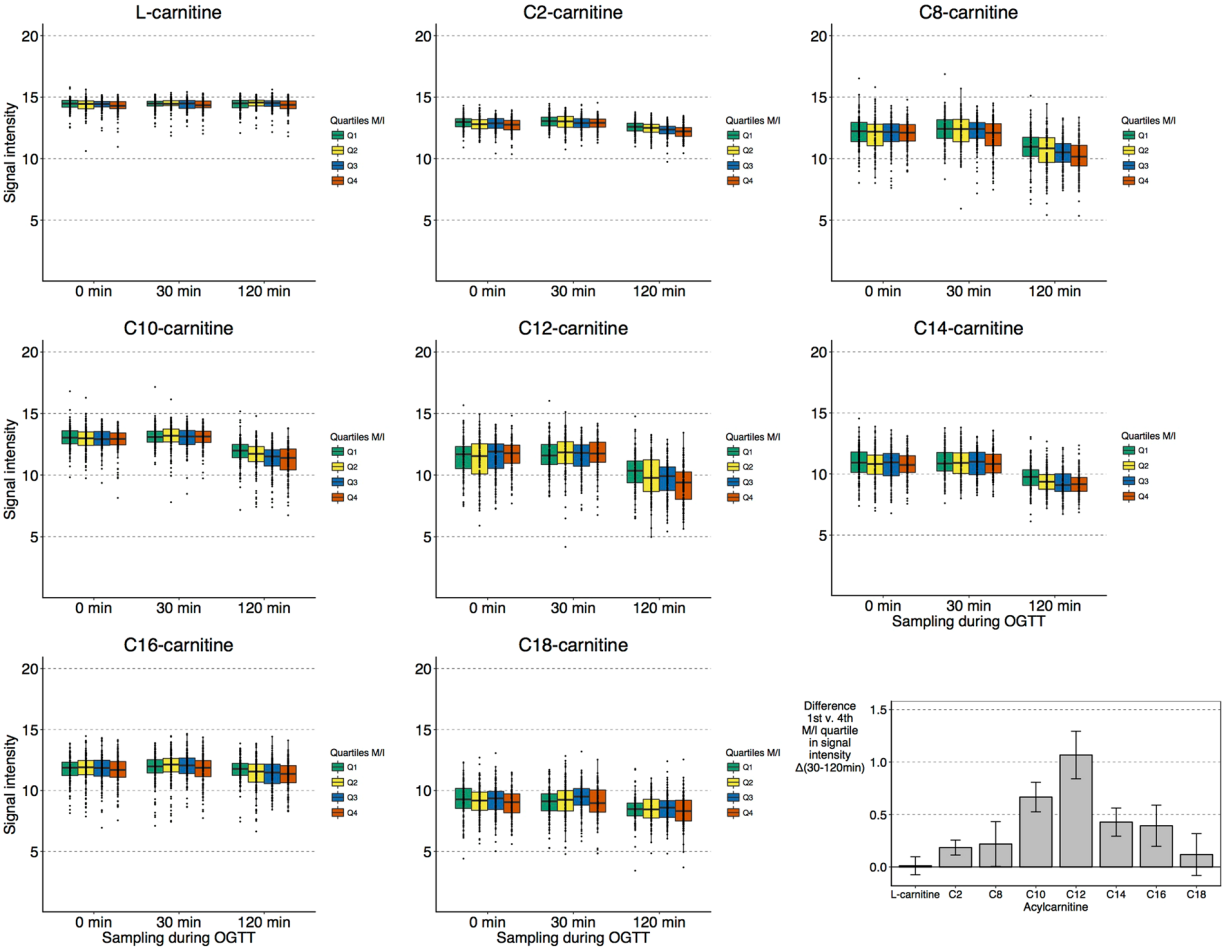
Abundance of plasma acylcarnitines during OGTT according to insulin sensitivity. (A) Log_2_-scaled, normalized UPLC-TOF-MS signal intensity of detected carnitine species according to quartile of insulin sensitivity (M/I) in boxplots (*n* = 470). (B) Difference in signal change 30–120 min between highest IR (M/I quartile 1, *n* = 117) and lowest IR (M/I quartile 4, *n* = 117) for different acylcarnitines.

The strongest associations with IR were observed for trajectories of C10-carnitine (*P* = 7.0 × 10^−7^ for Δ30–120 min) and C12-carnitine (*P* = 6.2 × 10^−8^ for Δ30–120 min). Additional adjustment for body mass index, fasting plasma glucose and fasting insulin somewhat attenuated these associations with maintained directions (Supplementary Table 3), as did additional adjustment for current smoking, physical exercise, alcohol intake and diet (daily intake of total energy, fat, protein and carbohydrates over a seven-day period; Supplementary Table 4). We assessed whether the associations with acylcarnitines were carbon chain length-specific, as longer chain-length has been associated with increasing IR^24,25^. Figure 2 shows levels of L-carnitine and all acylcarnitines that we were able to annotate using our untargeted metabolomics method (C2, C8, C10, C12, C14, C16 and C18, i.e. not including, for example, C3 and C5). Whilst all levels declined during OGTT, only C10- and C12-carnitine showed a significantly blunted decline in more insulin resistant individuals.

**Figure 3:**
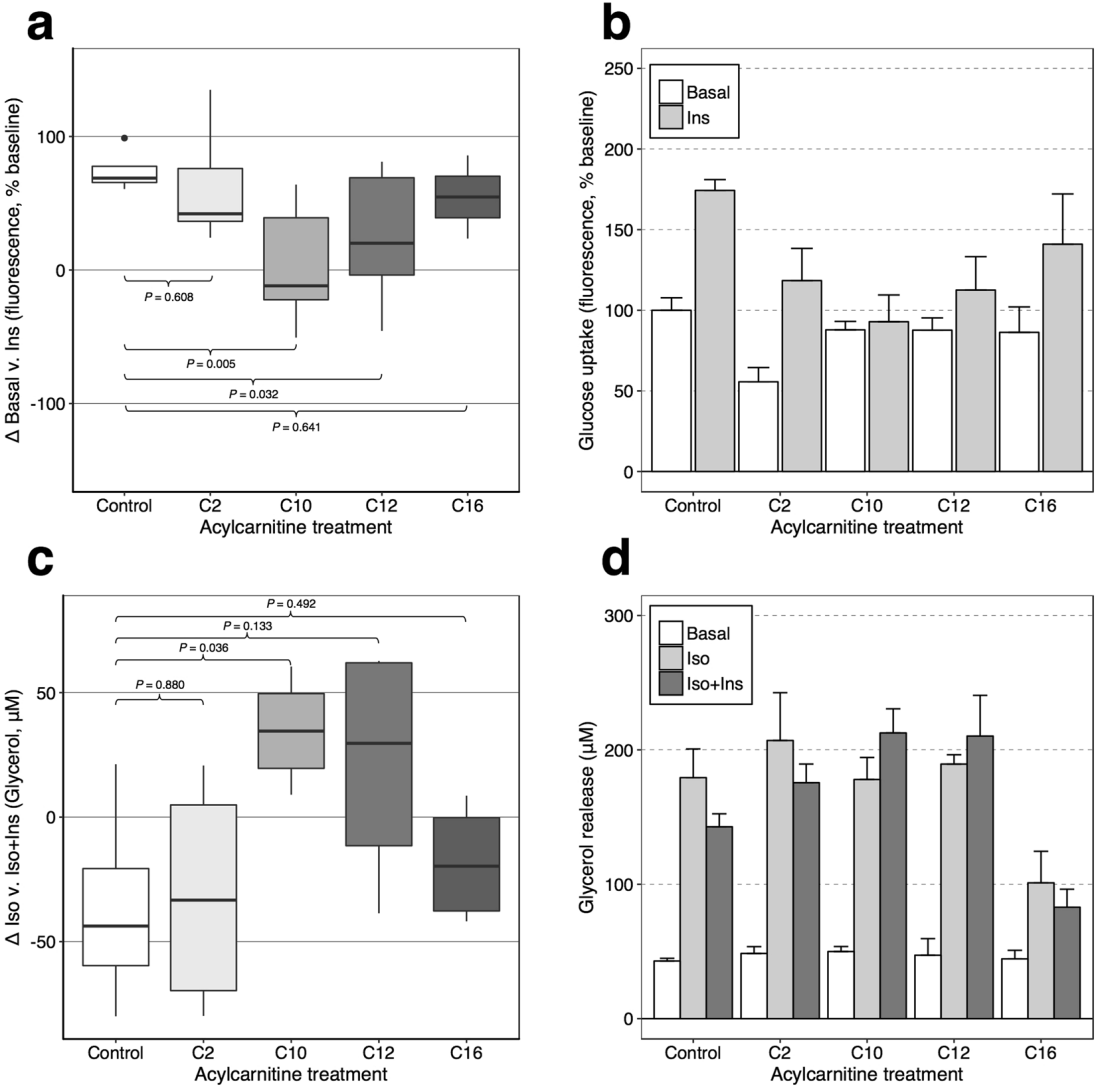
Glucose uptake and lipolysis in murine 3T3-L1 adipocytes. (**a,b**) Glucose uptake was quantified by fluorescent 2-NBD-glucose uptake (100 μg/mL, 20 min incubation with/without 100 nM insulin (Ins)) in DMEM (1 g/L glucose) following overnight serum-starvation and 2 h incubation with/without 100 µM acylcarnitines (including 1 h in glucose-free DMEM). Intracellular fluorescence is expressed in relation to basal uptake in the control cells. Differences in glucose uptake between basal and insulin-stimulated conditions are shown in boxplots (a); panel (b) shows individual conditions from the same experiments. (**c,d**) Lipolysis was measured in overnight serum-starved cells in DMEM over 1 h with/without exposure to 100 nM isoproterenol (Iso) ± 10 nM Ins. Cells were pre-incubated for 2 h with vehicle or 100 µM acylcarnitines. Cell density-normalized glycerol release is shown as the difference in glycerol release after insulin stimulation in panel (c), individual conditions in the same experiments are shown in panel (d). Experiments were reproduced in replicates for glucose uptake (n = 3 experiment) or triplicates for lipolysis (n = 2 per experiment) and combined results are shown. *P*-values are from two-tailed t-tests; error bars indicate standard errors.

Our findings appeared specific to C10 and C12 chain lengths and a distinct role of medium-chain acylcarnitines in IR has been suggested previously^25^. We therefore hypothesized that medium-chain acylcarnitines might impair insulin-stimulated glycemic response. We tested whether exposure to C2-, C10-, C12-, or C16-carnitine in murine 3T3-L1 adipocytes would affect insulin-stimulated glucose uptake (ISGU) and lipolysis. Based on the human results, we hypothesized that C10 and C12 would blunt insulin response, while short and long-chain acylcarnitines would not. Consistent with this hypothesis, we observed a reduction in ISGU after C10- and C12-carnitine incubation compared to control. The average (standard error) increase in glucose uptake after 100 nM insulin exposure was 74 ± 7% (control), 63 ± 20% (C2, *P*-value compared to control, *P* = 0.608), 5 ± 17% (C10, *P* = 0.005), 25 ± 21% (C12, *P* = 0.032), 55 ± 31% (C16, *P* = 0.641) (Fig. 3a,b). There was a consistent trend for impaired inhibition by insulin of isoproterenol-stimulated lipolysis after C10- or C12-carnitine incubation (Fig. 3c,d); effect of insulin on isoproterenol-stimulated glycerol release -20 ± 12% (control), -15 ± 12% (C2, *P* = 0.880), +19 ± 6% (C10, *P* = 0.036), +11 ± 13% (C12, *P* = 0.133), -18 ± 12% (C16, *P* = 0.492).

## Discussion

Our findings from the largest human sample assessed by OGTT metabolomics and intravenous measurement of insulin sensitivity, together with *in vitro* studies in murine adipocytes suggest a possible role of C10-carnitine and C12-carnitine in IR development. We further found significant differences in OGTT trajectories of monounsaturated FAs, several even-numbered LysoPEs and a bile acid between persons with more or less IR. Strengths of our study include a large sample assessed with these unique techniques, cohort homogeneity (decreasing the likelihood of confounding), rigorous quality control, and follow-up *in vitro*. The validity of our metabolomics pipeline is supported by the replication of reported OGTT metabolite trajectories including the overall decline of acylcarnitine^5,6,23^ and the expected association between IR and hexose. In contrast to metabolomics studies involving commercial platforms, we have made the data and methods publicly available. Limitations of our study include unknown generalizability to women and other age- and ethnic groups, and ~20 yr storage of plasma in −70 °C that may have affected measurements^20^. Validation in primary human adipocytes or skeletal muscle cells would have been preferable to the murine 3T3-L1 cell line, especially given the known biology of acylcarnitines. However, we did not have such cells available, and the 3T3-L1 adipocyte model is well established as a proxy for human IR^26,27^. Different acylcarnitines may be transported and metabolized differently, and therefore it is somewhat artificial to use the same concentration (100 μmol) in the *in vitro* studies. However, as there are no prior data on acylcarnitines in these systems, we chose this approach to standardize the experiments and to be able to compare results across acylcarnitines. Another limitation inherent to observational studies is the variability between participants with regards to diet, exercise and lifestyle factors that make it difficult to reach a precise picture of the underlying pathology without impact of potential confounder or reverse causation.

Our study contributes to clarifying the role of acylcarnitines in IR. Previous studies have suggested a particular role of medium-chain acylcarnitines in IR^24,25^, and our findings in the largest challenge study of its kind support their contribution. The observational link between overweight and IR notwithstanding^1^, adjustment for obesity had little effect on associations.

Plasma acylcarnitines may reflect cytosolic “overspill”, active cellular export, or may contribute to detoxification^28^. Our study cannot disentangle the origin and molecular effects of IR-associated acylcarnitines, but follow-up studies at the subcellular level may shed light on a possible primary dysfunction in FA oxidation. Genetic studies suggest predominant fat deposition in visceral over subcutaneous tissue as a more important contributor to IR than whole-body adiposity^29,30^. Limited sample size precluded us from testing for genetic effects. Nonetheless, we believe that our study provides important insights into the interplay between IR and aberrant FA oxidation. Even if the combination of human and cell experiments helps towards understanding the underlying mechanisms, we cannot definitively establish whether the associations between IR and medium-chain acylcarnitines reflect causal effects, incomplete FA oxidation, dysregulated competition between energy substrates, or other shared mechanisms.

The outer mitochondrial membrane enzyme carnitine palmitoyltransferase-1 (CPT1) catalyzes the conversion of FA-derived acyl-coenzyme A esters to acylcarnitines that are transported into the mitochondrion by carnitine acylcarnitine translocase. The mitochondrial matrix protein CPT2 then converts acylcarnitines back to acyl-coenzyme A esters for participation in beta-oxidation. In rodents, inhibition of both the skeletal muscle and the liver isoform of CPT1 has shown beneficial effects on insulin resistance^31,32^. Dysregulation of CPT with subsequent effects on energy production from FAs and impaired feedback regulation of glucose metabolism could underlie our observations on acylcarnitines. Our study cannot identify the molecular mechanisms linking C10 - and C12-carnitine to insulin resistance and future research may address these aspects in detail.

Previous small studies have demonstrated that acylcarnitines may contribute to IR through lipotoxicity related to dysfunctional beta-oxidation^33,34^, as well as through pro-inflammatory action^35^. Raised plasma concentrations of medium-chain acylcarnitines have been linked to increased CVD risk regardless of other risk factors, including branched chain amino acids and obesity. Interestingly, this association was absent in adults randomized to a Mediterranean diet intervention^36^. Alongside our findings on an adverse role in IR, the available evidence suggests medium-chain acylcarnitines as possible targets for cardiometabolic prevention. Given the protective effects of the Mediterranean diet for T2D and suggestive benefits for adverse acylcarnitine profiles^36^, the Mediterranean diet could provide a possible approach and further targeted research on a mediating role of medium-chain acylcarnitines for its effects are indicated.

Similarly to medium-chain acylcarnitines, the overall decline in concentrations of the monounsaturated FAs palmitoleate and oleate was blunted during the OGTT in persons with worse IR. Palmitoleate and oleate are derived from the diet and from endogenous production by the rate-limiting enzyme stearoyl CoA desaturase 1 (SCD-1)^37^. We previously reported genetic evidence for a concentration-lowering effect of IR on these two FAs in the fasting state and hypothesised a mediating role of SCD-1^12^. In addition to transcriptional regulation, the activity of SCD-1 is controlled through enzymatic and product/substrate regulation^38^. The blunted concentration decline of monounsaturated FAs in insulin resistant persons could be the result of reduced sensitivity of SCD-1 to intracellular signalling or impaired glucose metabolism and hence reduced production of SCD-1-inhibiting intermediates. Desensitized intracellular metabolic signalling may also underlie the OGTT trajectory association we observed for the bile acid-derivative deoxycholate-glycine, whose level rose in all participants in the early phase of the OGTT, albeit from a higher baseline level with a consequently flatter slope in more insulin resistant persons. Previous studies have demonstrated associations between elevated fasting concentrations of bile acids in the same chemical class as deoxycholate and IR as well as T2D^39,40^. In fasting samples, we recently confirmed the association between raised deoxycholate levels and risk of T2D, and identified a genetic polymorphism in *CYPA1* that was associated with reduced deoxycholate levels, raised LDL-cholesterol and reduced risk of T2D^13^. The findings in the metabolic challenge setting in the present study concur with the notion of an interaction of IR with bile acid metabolism predominantly in the fasted state, since OGTT levels rose and plateaued to similar levels in persons on both ends of the insulin resistance/insulin sensitivity spectrum. Ideally, however, our findings should be validated in human primary cells, in particular in skeletal muscle cells.

## Conclusion

In summary, nine out of 192 metabolite profiles were associated with IR, with the strongest signals detected for C10- and C12-carnitines, whose overall decline was blunted in more insulin resistant compared to insulin sensitive individuals. In mouse adipocytes, C10- and C12-carnitine incubation reduced insulin-stimulated glucose uptake, in line with a possible contributing mechanism for medium-chain acylcarnitines in IR development. In conclusion, we found that the decline in plasma levels of medium-chain acylcarnitines during a glucose challenge was blunted in IR, and demonstrated limited *in vitro* evidence for a contributing role of medium-chain acylcarnitines in impaired insulin-mediated glucose uptake.

## Electronic supplementary material

Supplementary Material
