# Supplementary material for: Glucose challenge metabolomics implicates medium-chain acylcarnitines in insulin resistance
Source: Sci Rep. 2018 Jun 6;8:8691. doi: 10.1038/s41598-018-26701-0 (PMC5989236; doi:10.1038/s41598-018-26701-0)
Supplement: Supplementary file 1 — Supplementary Material [file 41598_2018_26701_MOESM1_ESM.docx]

**Glucose challenge metabolomics implicates medium-chain acylcarnitines in insulin resistance**

Christoph Nowak, Susanne Hetty, Samira Salihovic, Casimiro Castillejo-Lopez, Andrea Ganna, Naomi L. Cook, Corey D. Broeckling, Jessica E. Prenni, Xia Shen, Vilmantas Giedraitis, Johan Ärnlöv, Lars Lind, Christian Berne, Johan Sundström, Tove Fall, Erik Ingelsson

**Supplementary Material**

**Supplemental Methods**

**Supplementary Fig. 1.** Study flow.

**Supplementary Fig. 2.** Product ion spectra for metabolites and corresponding standards (annotation level 1).

**Supplementary Fig. 3.** Product ion spectra for metabolites and corresponding standards (annotation level 2).

**Supplementary Fig. 4.** Product ion spectra for metabolites and corresponding standards (annotation level 3).

**Supplementary Fig. 5.** Relative change in metabolite levels during OGTT comparing men in the lowest and highest quartiles of insulin sensitivity.

**Supplementary Table 1.** Characteristics and association statistics of IR-related metabolites.

**Supplementary Table 2.** Association statistics for IR-related metabolite trajectories during OGTT.

**Supplementary Table 3.** Sensitivity analysis adjusting for potential confounders.

**Supplementary Table 4.** Sensitivity analysis adjusting top findings for dietary and lifestyle factors.

**Supplementary Table 5.** Replication of top results with total glucose disposal (M value) during clamp assessment as outcome (compare to Supplementary Table 2)

**Supplementary Methods**

***Ultraperformance liquid chromatography-time-of-flight mass spectrometry (UPLC-TOF-MS).*** Venous EDTA plasma samples were stored at to -70 °C. Samples were shipped for analysis by untargeted UPLC-TOF-MS to the Proteomics and Metabolomics Facility, Colorado State University, Fort Collins, CO, USA. Samples were thawed, underwent protein precipitation by mixing 100 μL sample with 400 μL methanol, kept overnight at -20 °C, and centrifuged for 30 min at 3,800 g and 4 °C. The supernatant was distributed across three 96-well plates that were covered with heat-seal foil and kept at -20 °C until analysis. Plates were analyzed in random sets of two, injections were randomized by plate order, and all samples were analyzed in duplicates. A Waters Acquity UPLC platform (Waters Corporation, Milford, MA, USA) was used to analyze 1 μL injections and instrument maintenance (cone cleaning, mass calibration, detector gain calibration and quality control injection) performed prior to running each pair of plates. The platform combines a Waters Acquity UPLC BEH C8 column (1.8 μM, 1 mm x 100 mm) prepared with a gradient from solvent A (95 % water, 5 % methanol, 0.1 % formic acid) to solvent B (95 % methanol, 5 % water, 0.1 % formic acid). Injections were performed in 100 % solvent A (held for 6 s), ramped to 40 % solvent B (54 s), 70 % solvent B (120 s) and 100 % solvent B (480 s). The mobile phase was kept at 100 % solvent B for 6 min, reset to starting conditions for 6 s and left to equilibrate for 5.9 min. A constant flow rate of 140 μL/min was used, the separation column maintained at 50 °C and the sample kept at 10 °C. The effluent was infused into a Waters Xevo G2-TOFMS fitted with an electrospray source and operated in positive ion mode at scanning range m/z 50 to 1,200 at a frequency of 5 Hz. Alternate scans at collision energies 6 V and 15-30 V were performed. Calibration prior to sample testing was implemented by sodium formate solution injection (mass accuracy 1 ppm). Capillary voltage was 2,200 V, the source temperature was 150 °C and desolvation was performed at 350 °C at a nitrogen gas flow rate of 800 L/h. A Quadrupole collision energy of 6 V was applied and DataBridge (Waters) software was used to export data as .cdf files.

***Metabolomics data processing.*** The source code of our bioinformatics pipeline is available for open access online (<https://github.com/andgan/metabolomics_pipeline/)> and has been described in detail previously.^1^ In brief, the *xcms* Bioconductor package^2^ in R was used for peak detection, peak alignment, peak grouping and peak imputation and parameters were optimized in simulations of 30 random samples (i.e. 120 chromatograms; for each individual, two replicates of MS and MS/MS). The selected parameterizations were quality-assessed in peak detection and peak grouping plots produced by *xcms*. Data were log-transformed and normalized by ordinary least squares linear regression fitted for metabolic feature intensity and covariates of unwanted variation (retention time correction, analysis date, sample collection, plate effects). Before further analysis, residuals were manually searched to exclude samples with aberrant feature intensities, low Spearman correlations between duplicate injections, or retention times < 35 s. Features were annotated according the Metabolomics Standards Initiative guidelines^3^ by matching m/z, retention time and spectral fragmentation pattern to an in-house reference library of standard compounds (Level 1), or to a specific metabolite (Level 2) or metabolite class (Level 3) available in METLIN (https://metlin.scripps.edu/)^4^ and HMBD (http://www.hmdb.ca/)^5^. Full raw metabolomics data are available by open access in the MetaboLights archive (http://www.ebi.ac.uk/metabolights/) under accession number MTBLS124. **Supplementary Figures 2 to 4** show product ion spectra for insulin resistance (IR) biomarkers obtained on our platform and the references used for annotation. **Supplementary Table 1** gives details on annotation level, m/z and retention time for IR-associated metabolites.

***Insulin sensitivity index M/I.*** The glucose disposal (M) was calculated as the amount of glucose taken up during the last 60 minutes of the study and is given in mg/kg bw/min. The insulin sensitivity index (M/I ratio) is a measure of the tissue sensitivity to insulin expressed per unit insulin and was calculated by dividing M by the mean insulin concentration during the same period of the clamp. M/I thus represents the amount of glucose metabolized per unit of plasma insulin and is given in mg/kg bw/min per mU/l of insulin multiplied by 100. The calculation of the total body insulin sensitivity is based on the assumption that endogenous hepatic glucose production is entirely suppressed. Under euglycaemic conditions it is known that almost 90% of this production is suppressed when the plasma insulin concentration is increased by 50 mU/L, as validated by Pollare et al 1991.^6^ Please see the online description of the ULSAM cohort for further details: http://www.pubcare.uu.se/ulsam/Database/ULSAM-70/methods#Z620. In our sample, the Pearson correlation between M value and M/I index was r = 0.9328. In the main analysis, we used the M/I as outcome (**Supplementary Table 2**). Re-analysis of the top associations using the M value as outcome essentially replicated the results (**Supplementary Table 5**).

**Supplementary Figure 1. Study flow.**


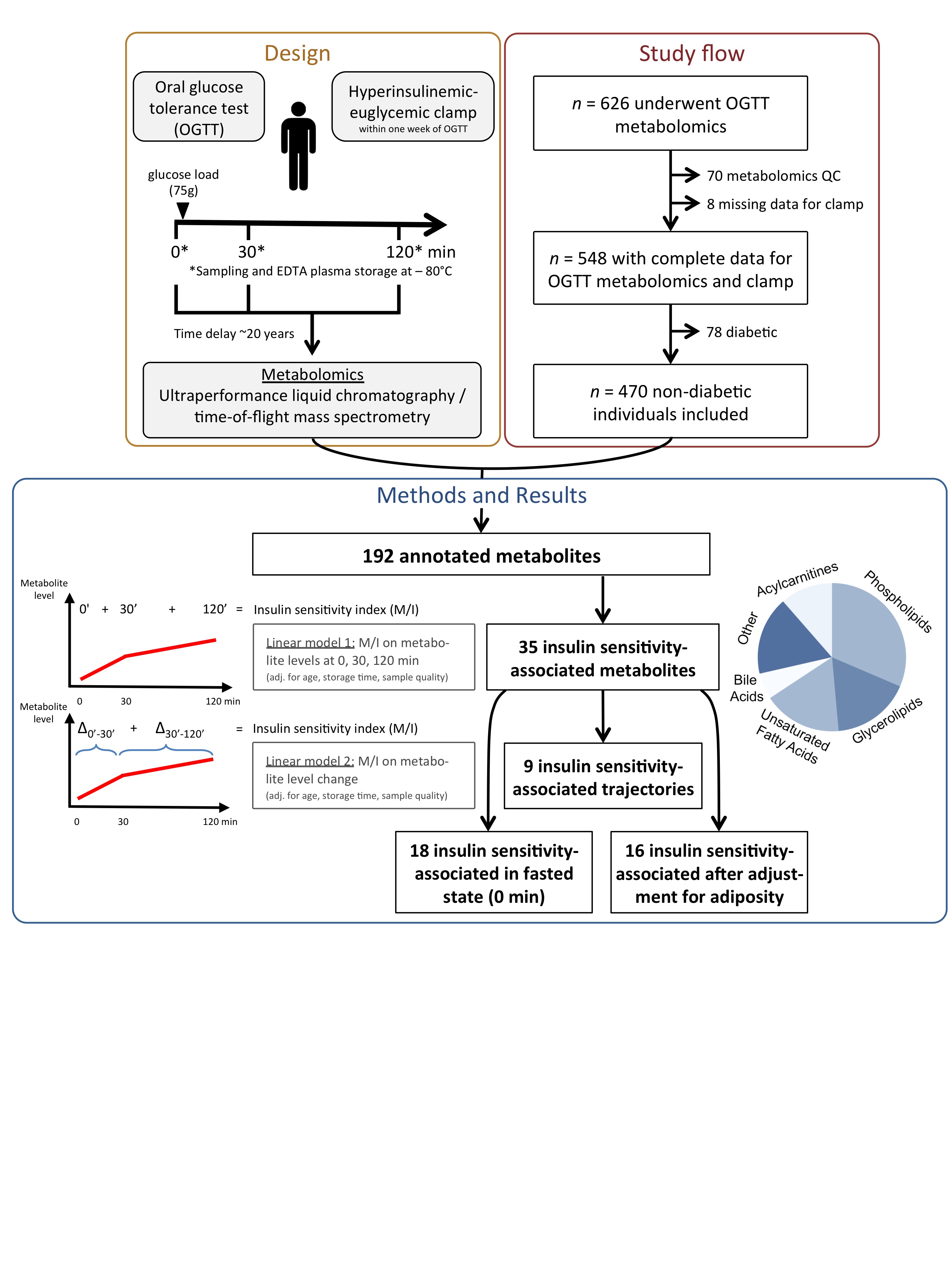


**Supplementary Figure 2.** Product ion spectra for metabolite features (upper spectrum) and corresponding standards (lower spectrum) using an ultraperformance liquid chromatography quadrupole time-of-flight mass spectrometer (UPLC-qTOF-MS) operated in positive electrospray ionization (ESI) mode; Metabolomics Standards Initiative (MSI, doi: 10.1007/s11306-007-0070-6) annotation confidence level 1.

**(A)** Metabolite eluting at 7.78 min represented by [2M + K]^+^ = 603.467 identified as oleic acid.

**(B)** Metabolite eluting at 6.62 min represented by [M + H]^+^ = 480.309 identified as lysophosphatidylethanolamine (LysoPE) (18:1).

**(C)** Metabolite eluting at 3.64 min represented by [M + H]^+^ = 344.280 identified as dodecanoylcarnitine.

**Supplementary Figure 3.** Product ion spectra and chemical structure of metabolites identified at MSI annotation confidence level 2.

**(A)** Metabolite identified as palmitoleic acid (C_16_H_30_O_2_, M = 254.224 Da) where the protonated molecule [M + H]^+^ = 255.232 and the adduct and fragment ions at m/z 293.3179, m/z 277.202, m/z 237.222, m/z 219.211, are represented by adduct formation [M + K]^+^ and [M + Na]^+^, as well as the loss of water [M + H - H_2_O] and [M + H - 2H_2_O].


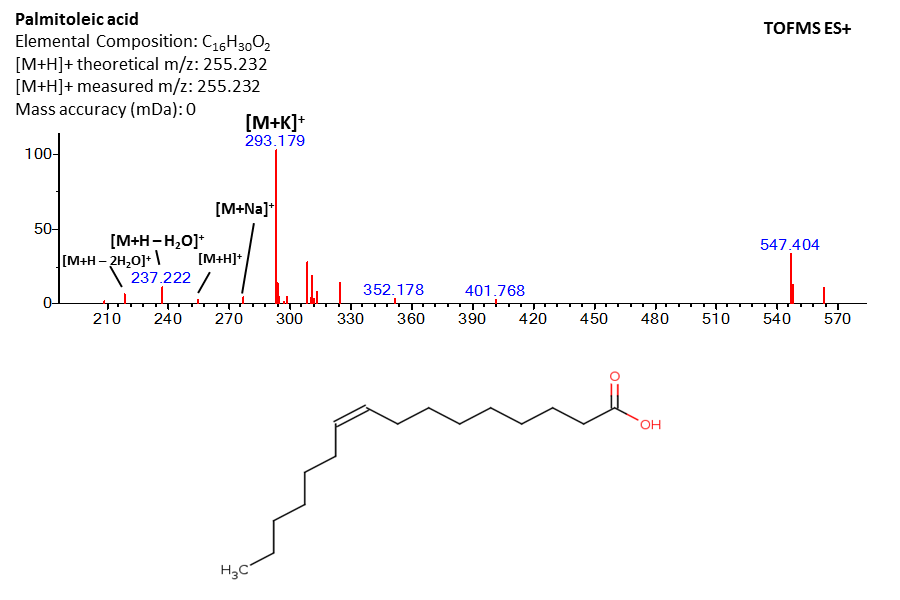


**(B)** Metabolite eluting at 7.78 min represented by [M + H]^+^ = 478.294 identified as LysoPE(18:2) .

**
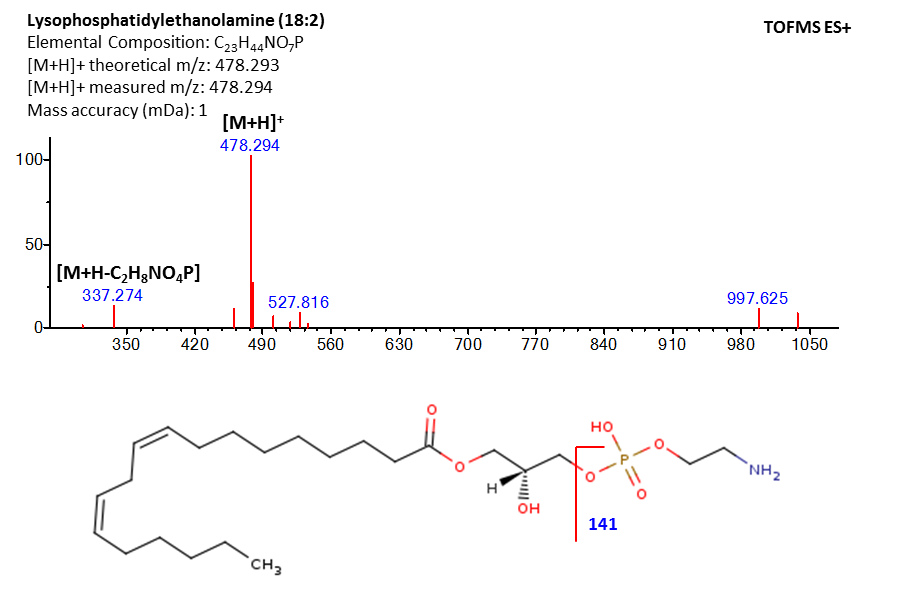
**

**(C)** Metabolite eluting at 6.07 min represented by [M + H]^+^ = 502.294 identified as LysoPE(20:4).

**
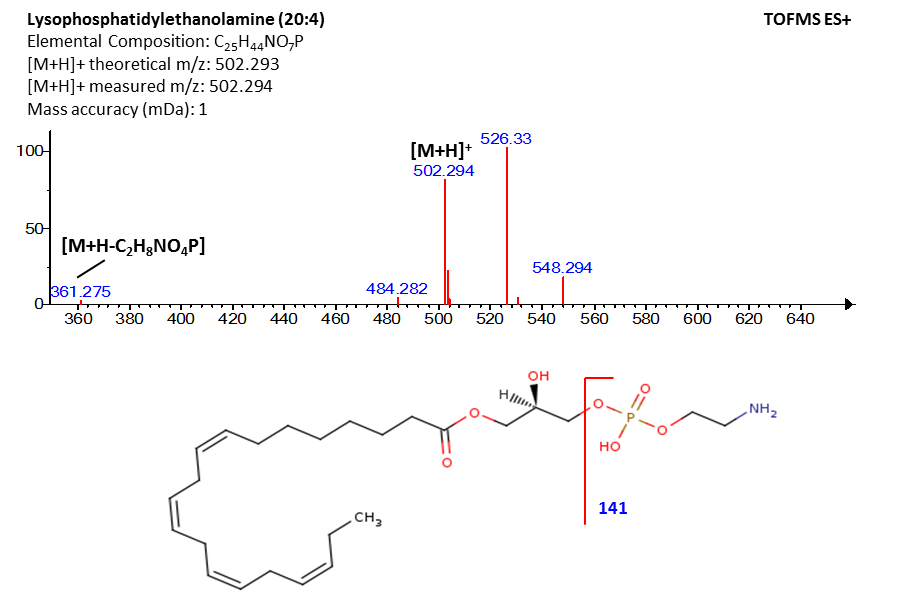
**

**(D)** Metabolite eluting at 3.12 min represented by [M + H]^+^ = 316.249 identified as decanoyl-L-carnitine.

**
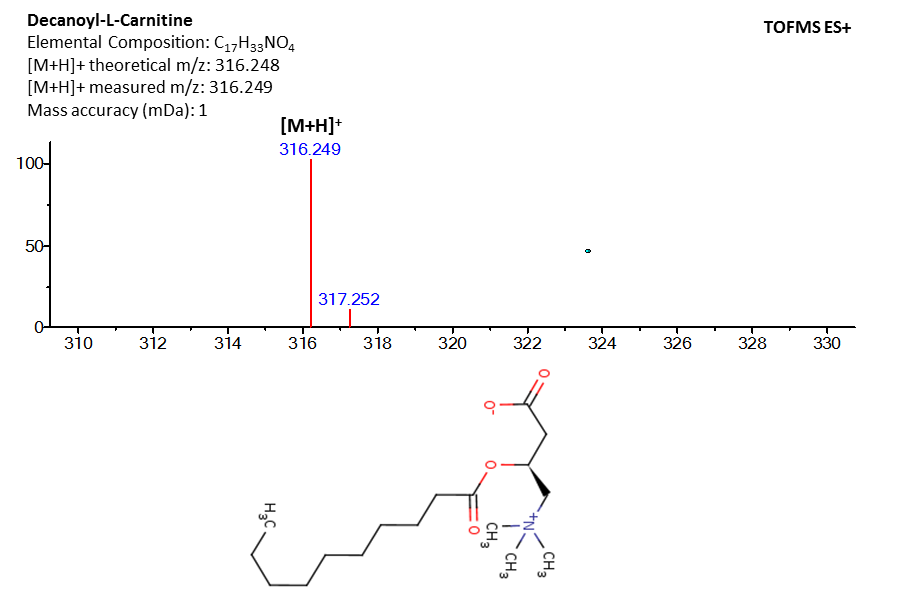
**

**Supplementary Figure 4**. Product ion spectra of metabolites annotated at MSI confidene level 3 obtained on a UPLC-qTOF-MS in positive ESI mode.

**(A)** Metabolite eluting at 3.91 min represented by [M + H – 2H_2_O]^+^ = 414.301 identified as deoxycholic acid-glycine conjugate.


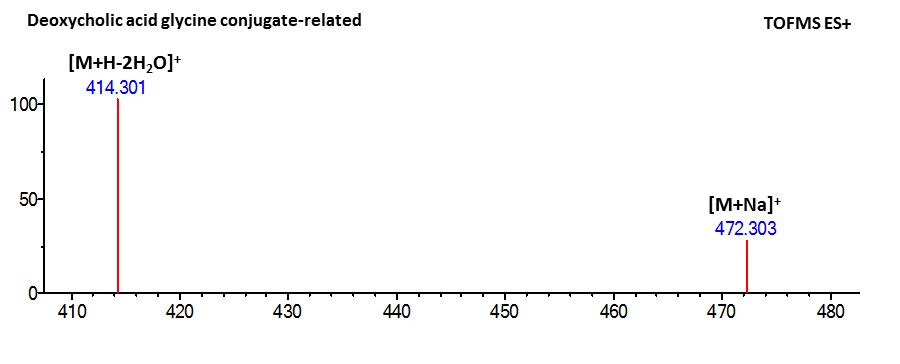


**(B)** Metabolite eluting at 0.55 min represented by [M + Na]^+^ = 203.053 identified as hexose.

**
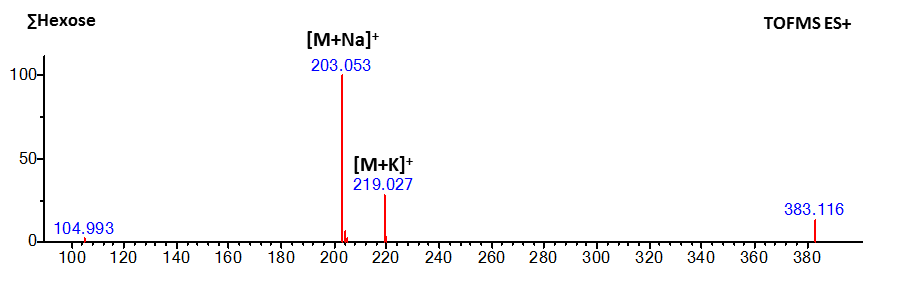
**

**Supplementary Figure 5. Relative change in metabolite levels during OGTT comparing men in the lowest and highest quartiles of insulin sensitivity.** Mean differences between three time points during OGTT test are shown for 35 metabolites associated with insulin sensitivity (IS). Age-adjusted metabolite signal intensities are scaled to SD-unit across the whole sample (*n* = 470). Change over time in the lowest (*n* = 117) and highest (*n* = 118) quartiles of insulin sensitivity is plotted on the x- and y-axis, respectively. Shapes and colors represent chemical classes. Symbol size is proportional to the *P*-value (-log_10_-transformed) of the regression coefficient representing the association between concentration change and insulin sensitivity (larger size indicates more significant association).

*Interpretation*: The centroid (0; 0) of each plot represents the average change across the entire sample for each comparison. Men with the worst IR are represented on the x-axis; men with the least IR on the y-axis. For instance, the change in dodecanoyl-carnitine (C12-carnitine) between 0-30 min (*left panel*) differs little between high and low IR - its mark is close to the intersection (0; 0). As shown in the non-transformed metabolite level plots in **Figure 1** of the main text, however, C12-carnitine declines less strongly from 30 to 120 min in individuals with high IR compared to a stronger decline in individuals with low IR. Hence, in the plot above (*middle panel*), the symbol for dodecanoyl-carnitine lies in the lower right quadrant, representing a *relative increase* in insulin resistant and a *relative decrease* in insulin sensitive individuals compared to overall sample mean.

**Supplementary Table 1. Characteristics and association statistics of 35 signficantly IR-related metabolites**

| **Metabolite** | **Annotation^A^** | **HMDB reference** | **Class** | **Overall linear regression model^B^** | | **LRT *P*-value compared to the baseline model adjusted for^B^** | | **Association at time points (*P*-values)^C^** | | |
| --- | --- | --- | --- | --- | --- | --- | --- | --- | --- | --- |
|  |  |  |  | **F statistic** | ***P*-value** | **age, sample quality** | **age, sample quality, BMI** | **0 min** | **30 min** | **120 min** |
| Monoacylglycerol 18:2 | 1 | HMDB11568 | Glycerolipid | 10.281 | 2.9 x 10^-13^ | 3.5 x 10^-11^ | 4.3 x 10^-8^ | 1.4 x 10^-9^* | 2.1 x 10^-6^* | 3.9 x 10^-11^* |
| Monoacylglycerol 18:1 | 1 | HMDB11536 |  | 9.361 | 5.3 x 10^-12^ | 7.7 x 10^-10^ | 2.2 x 10^-7^ | 1.2 x 10^-5^* | 9.3 x 10^-5^* | 1.5 x 10^-8^* |
| Monoacylglycerol 16:1 | 1 | HMDB11534 |  | 8.555 | 6.9 x 10^-11^ | 1.2 x 10^-8^ | 2.3 x 10^-4^ | 1.2 x 10^-5^* | 5.8 x 10^-3^ | 1.8 x 10^-9^* |
| Monoacylglycerol 16:0 | 1 | HMDB11533 |  | 7.804 | 7.6 x 10^-10^ | 1.5 x 10^-7^ | 9.6 x 10^-5^ | 1.9 x 10^-5^* | 2.4 x 10^-3^ | 1.7 x 10^-7^* |
| Monoacylglycerol 14:0 | 1 | HMDB11530 |  | 6.893 | 1.4 x 10^-8^ | 3.5 x 10^-6^ | 1.8 x 10^-3^ | 5.4 x 10^-4^* | 0.080 | 1.1 x 10^-6^* |
| 1-Vaccenoyl-2-palmitoyl-Sn-glycerol | 1 | HMDB07185 |  | 5.820 | 4.3 x 10^-7^ | 1.5 x 10^-4^ | 0.013 | 0.016 | 5.2 x 10^-3^ | 1.4 x 10^-4^* |
| 1-Arachidoyl-2-hydroxy-Sn-glycero-3-phosphocholine | 1 | [NA] | Glycero-phospholipid | 8.924 | 2.1 x 10^-11^ | 3.4 x 10^-9^ | 3.9 x 10^-4^ | 7.4 x 10^-7^* | 2.4 x 10^-7^* | 2.0 x 10^-6^* |
| 1-Oleoyl-2-hydroxy-Sn-glycero-3-phosphocholine | 1 | [NA] |  | 7.887 | 5.8 x 10^-10^ | 1.2 x 10^-7^ | 8.7 x 10^-4^ | 6.8 x 10^-6^* | 4.4 x 10^-5^* | 1.7 x 10^-6^* |
| Lysophosphatidylcholine 18:1a | 2 | [NA] |  | 6.870 | 1.5 x 10^-8^ | 3.8 x 10^-6^ | 7.6 x 10^-3^ | 2.3 x 10^-5^* | 5.5 x 10^-5^* | 8.6 x 10^-5^* |
| Lysophosphatidylcholine 18:2 | 1 | HMDB10386 |  | 6.716 | 2.5 x 10^-8^ | 6.5 x 10^-6^ | 0.024 | 5.7 x 10^-5^* | 1.3 x 10^-4^* | 2.0 x 10^-4^* |
| Lysophosphatidylcholine 18:3 | 2 | HMDB10388 |  | 6.594 | 3.6 x 10^-8^ | 1.0 x 10^-5^ | 6.2 x 10^-4^ | 6.0 x 10^-6^* | 3.0 x 10^-4^* | 5.0 x 10^-6^* |
| 1,2-Dilinoleoyl-Sn-glycero-3-phosphocholine | 1 | [NA] |  | 6.195 | 1.3 x 10^-7^ | 4.0 x 10^-5^ | 5.1 x 10^-4^ | 2.7 x 10^-5^* | 0.043 | 6.7 x 10^-4^* |
| Lysophosphatidylcholine 0:0/18:2 | 1 | [NA] |  | 5.926 | 3.1 x 10^-7^ | 1.0 x 10^-4^ | 0.104 | 2.5 x 10^-4^* | 4.7 x 10^-4^* | 1.5 x 10^-3^ |
| Lysophosphatidylethanolamine 18:2 | 2 | HMDB11507 | Glycero-phospho-ethanolamine | 10.044 | 6.1 x 10^-13^ | 7.7 x 10^-11^ | 2.5 x 10^-4^ | 2.3 x 10^-8^* | 1.3 x 10^-4^* | 0.379 |
| Lysophosphatidylethanolamine 18:1 | 2 | HMDB11505 |  | 8.655 | 5.0 x 10^-11^ | 8.4 x 10^-9^ | 1.1 x 10^-4^ | 2.1 x 10^-8^* | 2.9 x 10^-3^ | 0.344 |
| 1-Oleoyl-2-hydroxy-Sn-glycero-3-phosphoethanolamine | 1 | [NA] |  | 7.702 | 1.0 x 10^-9^ | 2.2 x 10^-7^ | 1.5 x 10^-4^ | 3.3 x 10^-4^* | 1.4 x 10^-6^* | 3.7 x 10^-7^* |
| Lysophosphatidylethanolamine 20:4 | 2 | HMDB11517 |  | 6.827 | 1.7 x 10^-8^ | 4.4 x 10^-6^ | 3.7 x 10^-3^ | 1.2 x 10^-5^* | 1.6 x 10^-3^ | 0.848 |
| Oleic acid | 1 | HMDB00207 | Unsaturated fatty acid | 9.685 | 1.9 x 10^-12^ | 2.6 x 10^-10^ | 2.3 x 10^-6^ | 0.153 | 1.2 x 10^-3^* | 2.7 x 10^-11^* |
| Palmitoleic acid | 2 | HMDB03229 |  | 7.915 | 5.3 x 10^-10^ | 1.0 x 10^-7^ | 1.2 x 10^-4^ | 0.261 | 9.5 x 10^-5^* | 4.7 x 10^-8^* |
| Eicosatrienoic acid | 3 | [NA] |  | 7.771 | 8.4 x 10^-10^ | 1.7 x 10^-7^ | 2.0 x 10^-4^ | 1.5 x 10^-3^ | 1.3 x 10^-6^* | 9.3 x 10^-5^* |
| Linoleic acid | 1 | HMDB00673 |  | 6.600 | 3.6 x 10^-8^ | 9.7 x 10^-6^ | 9.9 x 10^-5^ | 0.135 | 2.6 x 10^-5^* | 4.9 x 10^-5^* |
| Alpha-linolenic acid | 1 | HMDB01388 |  | 6.265 | 1.0 x 10^-7^ | 3.1 x 10^-5^ | 4.1 x 10^-3^ | 0.102 | 6.6 x 10^-4^* | 4.8 x 10^-6^* |
| Arachidonic acid ethyl ester | 2 | [NA] |  | 6.258 | 1.1 x 10^-7^ | 3.2 x 10^-5^ | 9.4 x 10^-4^ | 1.5 x 10^-3^ | 1.5 x 10^-4^* | 7.8 x 10^-5^* |
| Dodecanoylcarnitine | 1 | HMDB02250 | Acylcarnitine | 7.933 | 5.0 x 10^-10^ | 9.8 x 10^-8^ | 8.8 x 10^-7^ | 0.458 | 0.967 | 1.5 x 10^-7^* |
| Decanoyl-L-carnitine | 2 | HMDB00651 |  | 7.526 | 1.8 x 10^-9^ | 4.0 x 10^-7^ | 1.5 x 10^-4^ | 0.047 | 0.426 | 1.2 x 10^-7^* |
| L-Acetylcarnitine | 1 | HMDB00201 |  | 6.331 | 8.4 x 10^-8^ | 2.5 x 10^-5^ | 2.3 x 10^-3^ | 0.085 | 0.049 | 1.1 x 10^-6^* |
| L-Octanoylcarnitine | 2 | HMDB00791 |  | 5.936 | 3.0 x 10^-7^ | 9.7 x 10^-5^ | 1.9 x 10^-3^ | 0.315 | 1.7 x 10^-3^ | 6.4 x 10^-5^* |
| Deoxycholic acid glycine conjugate | 1 | HMDB00631 | Bile acid | 5.862 | 3.8 x 10^-7^ | 1.3 x 10^-4^ | 2.1 x 10^-3^ | 1.7 x 10^-5^* | 8.7 x 10^-3^ | 0.108 |
| Deoxycholic acid glycine conjugate related metabolite | 3 | [NA] |  | 5.741 | 5.5 x 10^-7^ | 1.9 x 10^-4^ | 4.9 x 10^-5^ | 0.436 | 0.196 | 0.012 |
| Hexose | 3 | [NA] | Monosaccharide | 10.307 | 2.7 x 10^-13^ | 3.2 x 10^-11^ | 1.8 x 10^-9^ | 0.026 | 6.9 x 10^-4^* | 5.9 x 10^-12^* |
| Propranolol | 1 | HMDB01849 | Naphthalene | 7.853 | 6.5 x 10^-10^ | 1.3 x 10^-7^ | 1.3 x 10^-4^ | 1.9 x 10^-3^ | 1.2 x 10^-5^* | 2.0 x 10^-8^* |
| Uric acid | 1 | HMDB00289 | Imidazopyrimidine | 6.383 | 7.1 x 10^-8^ | 2.1 x 10^-5^ | 0.161 | 0.017 | 4.9 x 10^-6^* | 0.010 |
| Myristic acid | 1 | HMDB00806 | Saturated fatty acid | 5.744 | 5.5 x 10^-7^ | 1.9 x 10^-4^ | 3.1 x 10^-3^ | 0.015 | 0.159 | 1.5 x 10^-4^* |
| Gamma-glutamyl-leucine | 2 | HMDB29153 | Peptide | 5.727 | 5.8 x 10^-7^ | 2.0 x 10^-4^ | 0.289 | 3.1 x 10^-3^ | 5.7 x 10^-5^* | 8.2 x 10^-4^* |
| Corticosterone | 1 | HMDB01547 | Steroid | 5.677 | 6.8 x 10^-7^ | 2.4 x 10^-4^ | 0.016 | 3.2 x 10^-5^* | 1.9 x 10^-3^ | 2.0 x 10^-3^ |

^A^Accuracy according to the Metabolomics Standard Initiative .

^B^Linear regression combining metabolite levels at 0, 30, 120 min, age, storage time, previous thawing, sample type, and possible hemolysis as covariates; LRT compare the full to the reduced model excluding metabolites.

^C^*P*-values for the association of metabolite levels with IS at individual time points adjusted for age and sample quality parameters derived from linear regression models.

*Indicates significant associations at the Bonferroni-corrected alpha threshold.

Underlined values indicate positive associations with insulin sensitivity.

HMDB, the Human Metabolome Database; LRT, likelihood ratio test.

**Supplementary Table 2. Association statistics for IR-related metabolite trajectories during OGTT**

| **Metabolite** | **F-statistic *P*-value** | **Change 0-30 min** | | **Change 30-120 min** | |
| --- | --- | --- | --- | --- | --- |
|  |  | **β ± SEM** | ***P*-value** | **β ± SEM** | ***P*-value** |
| LysoPE 18:1 | 1.2 x 10^-5^ | -1.06 ± 0.25 | 2.5 x 10^-5^ | -0.87 ± 0.24 | 2.6 x 10^-4^ |
| LysoPE 18:2 | 7.3 x 10^-8^ | -1.14 ± 0.28 | 5.0 x 10^-5^ | -1.48 ± 0.28 | 1.7 x 10^-7^ |
| LysoPE 20:4 | 3.0 x 10^-4^ | -0.55 ± 0.21 | 8.3 x 10^-3^ | -0.78 ± 0.21 | 1.5 x 10^-4^ |
| C12-carnitine | 3.4 x 10^-7^ | -0.24 ± 0.07 | 8.9 x 10^-4^ | -0.36 ± 0.07 | 6.2 x 10^-8^ |
| C10-carnitine | 2.0 x 10^-6^ | -0.09 ± 0.13 | 0.466 | -0.58 ± 0.10 | 7.0 x 10^-7^ |
| Oleic Acid | 3.3 x 10^-5^ | -0.31 ± 0.11 | 6.9 x 10^-3^ | -0.42 ± 0.10 | 1.5 x 10^-5^ |
| Palmitoleic acid | 1.7 x 10^-4^ | -0.41 ± 0.13 | 1.7 x 10^-3^ | -0.33 ± 0.12 | 7.8 x 10^-3^ |
| Deoxycholic acid-glycine | 1.2 x 10^-4^ | 0.54 ± 0.13 | 4.7 x 10^-5^ | 0.41 ± 0.12 | 1.1 x 10^-3^ |
| Hexose | 3.8 x 10-5 | -0.61 ± 0.29 | 0.037 | -1.16 ± 0.26 | 8.8 x 10^-6^ |

Based on linear regression models of clamp M/I = (M_30_-M_0_) + (M_120_-M_30_); where M_0_, M_30_, and M_120_ represent metabolite levels at 0, 30, and 120 min adjusted for age and sample quality parameters. Only metabolites associated at the Bonferroni-corrected significance threshold (*P* < 1.43 x 10^-3^) are shown.

**Supplementary Table 3. Sensitivity analysis adjusting for potential confounders**

| **Metabolite and adjustments** | **Change 0-30 min** | | **Change 30-120 min** | |
| --- | --- | --- | --- | --- |
|  | **β ± SEM** | ***P*-value** | **β ± SEM** | ***P*-value** |
| C-10 carnitine | -0.09 ± 0.13 | 0.466 | -0.58 ± 0.10 | 7.0 x 10^-7^ |
| C-12 carnitine | -0.24 ± 0.07 | 8.9 x 10^-4^ | -0.36 ± 0.07 | 6.2 x 10^-8^ |
| C-10 carnitine, adj. FG | -0.08 ± 0.12 | 0.490 | -0.48 ± 0.10 | 8.9 x 10^-7^ |
| C-12 carnitine, adj. FG | -0.22 ± 0.07 | 1.0 x 10^-3^ | -0.33 ± 0.06 | 9.3 x 10^-8^ |
| C-10 carnitine, adj. FI | -0.12 ± 0.11 | 0.291 | -0.36 ± 0.09 | 9.3 x 10^-5^ |
| C-12 carnitine, adj. FI | -0.19 ± 0.06 | 1.9 x 10^-3^ | -0.28 ± 0.06 | 1.3 x 10^-6^ |
| C-10 carnitine, adj. BMI | -0.06 ± 0.11 | 0.582 | -0.42 ± 0.09 | 1.8 x 10^-6^ |
| C-12 carnitine, adj. BMI | -0.12 ± 0.06 | 4.4 x 10^-2^ | -0.21 ± 0.06 | 2.6 x 10^-4^ |
| C-10 carnitine, adj. BMI_obese | -0.07 ± 0.12 | 0.553 | -0.47 ± 0.10 | 1.5 x 10^-6^ |
| C-12 carnitine, adj. BMI_obese | -0.23 ± 0.07 | 5.5 x 10^-4^ | -0.32 ± 0.06 | 2.0 x 10^-7^ |
| C-10 carnitine, adj. FG, FI, BMI | -0.08 ± 0.10 | 0.436 | -0.33 ± 0.08 | 6.5 x 10^-5^ |
| C-12 carnitine, adj. FI, FG, BMI | -0.12 ± 0.06 | 3.4 x 10^-2^ | -0.19 ± 0.05 | 3.7 x 10^-4^ |

Based on linear regression models of clamp M/I = (M_30_-M_0_) + (M_120_-M_30_); where M_0_, M_30_, and M_120_ represent metabolite levels at 0, 30, and 120 min adjusted for age and sample quality parameters. Additional adjustments were fasting plasma glucose (FG, in mmol/L), fasting plasma insulin (FI, in mU/L), body mass index (BMI, in kg/m^2^), and dummy for BMI in the obese range (BMI_obese, > 30 kg/m^2^ vs. ≤ 30 kg/m^2^, *n* = 51 out of 470 had BMI > 30 kg/m^2^).

**Supplementary Table 4. Sensitivity analysis adjusting top findings for dietary and lifestyle factors.** Analyses are adjusted for current smoking (77 yes / 393 no), "at least 3 hr of physical activity per week" (281 yes / 189 no), "regular hard physical exercise" (30 yes / 440 no), "how often do you drink alcoholic beverages" (11.3% never, 27.2% seldom, 19.3% 1-2/month, 24.3% 1/week, 13.0% 2-3/week, 1.2% every day) and the following values calculated from a 7-day food diary: total daily energy intake (mean 1726.3 kcal/day, SD 493.3), daily fat intake (68.6 g/day, SD 24.4), daily protein intake (64.5 g/day, SD 18.0), and daily carbohydrate intake (204.5 g/day, SD 62.9).

| **Metabolite** | **F-statistic *P*-value** | **Change 0-30 min** | | **Change 30-120 min** | |
| --- | --- | --- | --- | --- | --- |
|  |  | **β ± SEM** | ***P*-value** | **β ± SEM** | ***P*-value** |
| LysoPE 18:1 | 2.8 x 10^-5^ | -1.08 ± 0.25 | 2.6 x 10^-5^ | -0.79 ± 0.24 | 1.0 x 10^-3^ |
| LysoPE 18:2 | 7.4 x 10^-7^ | -1.22 ± 0.29 | 2.7 x 10^-5^ | -1.31 ± 0.29 | 6.3 x 10^-6^ |
| LysoPE 20:4 | 1.3 x 10^-4^ | -0.70 ± 0.21 | 1.2 x 10^-3^ | -0.78 ± 0.21 | 2.0 x 10^-4^ |
| C12-carnitine | 4.0 x 10^-5^ | -0.20 ± 0.07 | 5.3 x 10^-3^ | -0.31 ± 0.07 | 8.3 x 10^-6^ |
| C10-carnitine | 1.8 x 10^-5^ | -0.08 ± 0.13 | 0.550 | -0.48 ± 0.10 | 6.4 x 10^-6^ |
| Oleic Acid | 3.0 x 10^-5^ | -0.31 ± 0.11 | 7.1 x 10^-3^ | -0.42 ± 0.10 | 1.3 x 10^-5^ |
| Palmitoleic acid | 2.0 x 10^-3^ | -0.40 ± 0.13 | 2.7 x 10^-3^ | -0.35 ± 0.13 | 6.0 x 10^-3^ |
| Deoxycholic acid-glycine | 4.3 x 10^-4^ | 0.48 ± 0.13 | 4.3 x 10^-4^ | 0.43 ± 0.13 | 6.3 x 10^-4^ |
| Hexose | 9.7 x 10^-5^ | -0.67 ± 0.30 | 0.024 | -0.94 ± 0.27 | 4.5 x 10^-4^ |

**Supplementary Table 5.** Replication of top results with total glucose disposal (M value) during clamp assessment as outcome (compare to Supplementary Table 2)

| **Metabolite** | **F-statistic *P*-value** | **Change 0-30 min** | | **Change 30-120 min** | |
| --- | --- | --- | --- | --- | --- |
|  |  | **β ± SEM** | ***P*-value** | **β ± SEM** | ***P*-value** |
| LysoPE 18:1 | 1.3 x 10^-5^ | -1.05 ± 0.25 | 3.2 x 10^-5^ | -0.88 ± 0.24 | 2.3 x 10^-4^ |
| LysoPE 18:2 | 8.4 x 10^-8^ | -1.13 ± 0.28 | 6.6 x 10^-5^ | -1.48 ± 0.28 | 1.7 x 10^-7^ |
| LysoPE 20:4 | 2.2 x 10^-4^ | -0.59 ± 0.21 | 5.9 x 10^-3^ | -0.79 ± 0.21 | 1.3 x 10^-4^ |
| C12-carnitine | 3.6 x 10^-7^ | -0.23 ± 0.07 | 9.6 x 10^-4^ | -0.36 ± 0.08 | 6.6 x 10^-8^ |
| C10-carnitine | 1.8 x 10^-6^ | -0.09 ± 0.13 | 0.471 | -0.52 ± 0.10 | 6.2 x 10^-7^ |
| Oleic Acid | 2.7 x 10^-5^ | -0.31 ± 0.11 | 6.0 x 10^-3^ | -0.42 ± 0.10 | 1.3 x 10^-5^ |
| Palmitoleic acid | 1.4 x 10^-3^ | -0.42 ± 0.13 | 1.5 x 10^-3^ | -0.33 ± 0.12 | 7.2 x 10^-3^ |
| Deoxycholic acid-glycine | 1.2 x 10^-4^ | 0.54 ± 0.13 | 4.6 x 10^-5^ | 0.41 ± 0.12 | 1.0 x 10^-3^ |
| Hexose | 3.2 x 10^-5^ | -0.62 ± 0.29 | 0.034 | -1.17 ± 0.27 | 7.3 x 10^-6^ |

**References**

1 Ganna, A. *et al.* Large-scale non-targeted metabolomic profiling in three human population-based studies. *Metabolomics* **12**, 1-13, doi:10.1007/s11306-015-0893-5 (2015).

2 Smith, C. A., Want, E. J., O'Maille, G., Abagyan, R. & Siuzdak, G. XCMS: processing mass spectrometry data for metabolite profiling using nonlinear peak alignment, matching, and identification. *Analytical chemistry* **78**, 779-787, doi:10.1021/ac051437y (2006).

3 Sumner, L. W. *et al.* Proposed minimum reporting standards for chemical analysis Chemical Analysis Working Group (CAWG) Metabolomics Standards Initiative (MSI). *Metabolomics : Official journal of the Metabolomic Society* **3**, 211-221, doi:10.1007/s11306-007-0082-2 (2007).

4 Smith, C. A. *et al.* METLIN: a metabolite mass spectral database. *Therapeutic drug monitoring* **27**, 747-751 (2005).

5 Wishart, D. S. *et al.* HMDB: the Human Metabolome Database. *Nucleic acids research* **35**, D521-526, doi:10.1093/nar/gkl923 (2007).

6 Pollare, T., Vessby, B. & Lithell H.. Lipoprotein activity in skeletal muscle is related to insulin sensitivity. *Arteriosclerosis and thrombosis* **11**(5), 1192-203 (1991)
